# A New Geo-Propagation Model of Event Evolution Chain Based on Public Opinion and Epidemic Coupling

**DOI:** 10.3390/ijerph17249235

**Published:** 2020-12-10

**Authors:** Yan Zhang, Nengcheng Chen, Wenying Du, Shuang Yao, Xiang Zheng

**Affiliations:** 1 State Key Laboratory of Information Engineering in Surveying, Mapping, and Remote Sensing, Wuhan University, Wuhan 430079, China; sggzhang@whu.edu.cn (Y.Z.); cnc@whu.edu.cn (N.C.); yaoshuang@whu.edu.cn (S.Y.); 2 School of Information Management, Wuhan University, Wuhan 430079, China; zhengxiang059@whu.edu.cn

**Keywords:** complex network, community structure, coupling evolution, barycenter migration, gravitation model, COVID-19

## Abstract

The online public opinion is the sum of public views, attitudes and emotions spread on major public health emergencies through the Internet, which maps out the scope of influence and the disaster situation of public health events in real space. Based on the multi-source data of COVID-19 in the context of a global pandemic, this paper analyzes the propagation rules of disasters in the coupling of the spatial dimension of geographic reality and the dimension of network public opinion, and constructs a new gravity model-complex network-based geographic propagation model of the evolution chain of typical public health events. The strength of the model is that it quantifies the extent of the impact of the epidemic area on the surrounding area and the spread of the epidemic, constructing an interaction between the geographical reality dimension and online public opinion dimension. The results show that: The heterogeneity in the direction of social media discussions before and after the “closure” of Wuhan is evident, with the center of gravity clearly shifting across the Yangtze River and the cyclical changing in public sentiment; the network model based on the evolutionary chain has a significant community structure in geographic space, divided into seven regions with a modularity of 0.793; there are multiple key infection trigger nodes in the network, with a spatially polycentric infection distribution.

## 1. Introduction

China adopts active prevention and control measures to effectively control the development of the Corona Virus Disease 2019 (COVID-19) pandemic [1]. On 22 January 2020, the number of diagnosed cases in Hubei province exceeded 100 in a single day and Hubei Province launched a level II public health emergency response. On 23 January 2020, at 10:00 a.m., Wuhan was under lockdown. Its public transportation such as subways, ferries, long-distance passenger were suspended, and the airport and railway stations were temporarily closed. On 24 January 2020, a total of 830 confirmed cases have been reported nationwide and Hubei Province launched the level I response to public health emergencies. Since then, medical teams all over the country have been sent to aid Hubei, and Wuhan has become the focus of people’s concern. After 76 days of active anti-epidemic efforts, Wuhan announced on 8 April 2020 that the control of the exit channel from Wuhan was lifted (www.xinhuanet.com/mrdx/2020-04/08/c_138956757.htm), and the prevention and control situation changed positively for the better and achieved important stage results.

In view of the fact that the basic reproductive number ($R_{0}$) of this pandemic event is higher than that of “SARS”, it poses a challenge to the development of containment of the pandemic [2]. With the increasing popularity of wireless network and social media activities, multiple types and multi-dimensional data will be generated in the comprehensive cycle of breeding, outbreak, response, and recovery of major infectious diseases. Some scholars have conducted the interaction between virus and environment [3], risk assessment from the geographical perspective [4], telemedicine [5], virus transmission and epidemic prediction [6,7], etc. studies on these data.

During the epidemic period, social media represented by Sina Weibo and Twitter became an important platform for residents to obtain information and express their opinions [8,9]. Social media is regarded as the representative of volunteered geographic information (VGI) and contains a variety of interaction patterns [10]. Many researchers mine more public security event knowledge based on these patterns for disaster response and damage assessment [11]. However, the common interactions in social media, including forwarding, liking, and so on, are usually not geotagged [12]. To solve this problem, check-in data with high precision coordinates were adopted by researchers [13,14].

Based on the interdisciplinary concept of “social geographic calculation” [15,16], this paper attempts to introduce geographic location information into the analysis of social media public opinion, then mine public opinion from the spatial dimension. Some scholars have studied the environmental perception and urban calculation of major emergency based on VGI data, surveyed a sample of Twitter messages under the earthquake disaster, and conducted research in the areas of geographic entity identification [17], detection of regional anomalies [18], analysis of the happiness level of different cities [19], and determination of public attitudes [13]. In addition, Cross regional migration of population [20] and seeking help on social media [21] have also been applied and discussed during this pandemic. These studies have broadened social media public opinion research ideas and fully proved that geospatial information plays an indispensable role in social media public opinion analysis.

**Figure 1:**
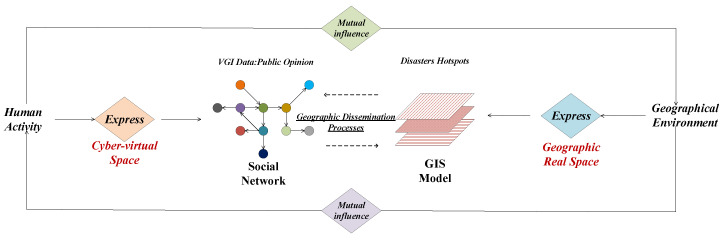
The coupling effect of cyberspace and geospatial.

Information such as the content, time, and location of residents’ blog posted on social media not only reflects their activities, psychological states, and hotspots of concern, but also mirrors the impact and infections in different communities during the epidemic period. Taking into account the influence of specific geographic regions on the evolution of public opinion and citizens’ emotions. As shown in Figure 1, this paper introduces geographic location information into the analysis of public opinion, combines traditional public opinion analysis methods with geographic calculations. In the context of the global influenza pandemic (www.cdc.gov/flu/pandemic-resources), based on the social network information (Sina Weibo) and geospatial information (community infection, road network, fever clinic) during the epidemic period as the object of study, the interaction analysis is conducted in time, space, and content levels [22,23,24].

Based on the social geographic calculation perspective, this paper proposes a new approach to the study of this pandemic based on complex networks. A complex network is a large-scale network with a complex structure, a topological abstract expression of the real system and somewhere between random and regular networks [25]. Its nodes can be arbitrary basic units with specific information content. For traditional “neatly aligned” Euclidean Structure Data, they can be clearly represented as a grid type, where any point in the grid can easily find its neighbor nodes, which can be achieved with classical neural networks (CNN, RNN). In real life, in addition to “regular” grid structure data, there are Non-Euclidean Structure Data such as social networks, compound structures, biogenetic proteins, and knowledge graphs. A point in Non-Euclidean structural data is difficult to define its neighbor nodes and the number of neighbor nodes is different for different nodes. In addition, complex networks have some concepts such as the small-world effect, degree distributions, clustering, network correlations, random graph models, models of network growth and preferential attachment, and dynamical processes taking place on networks [26]. Research on studying coupled disease–behavior dynamics in complex networks is growing rapidly [27], and it has many applications in the study of the COVID-19 pandemic. Simulation results for complex network methods show that it is possible to cut off connections between symptomatically infected individuals and their neighbors, and cut off connections between hub nodes and their neighbors [28]. Some authors treat airports as network nodes and air traffic flows as weights of complex networks, but do not consider the spatial interactions between nodes [29]. At the same time, some scholars have used gravity models to predict infection [30], suggesting that epidemics are spatially linked and exhibit an inverse square relationship [31]. We believe that it is appropriate to consider the influence of the nodes on the surroundings and to combine complex networks with gravitational models.

We take the COVID-19 epidemic event as the background to further understand the magnitude and distribution of the spatial impact of this major public security event, and construct a spatial impact evaluation model of the epidemic based on the reciprocal relationship between network activities and the geographic environment, including users’ network activities such as check-in information, concern information, interaction information, emotional information. This will help to quantify the spatially coupled impact of public opinion and disasters, detect epidemic hotspots, refine the geographic dissemination processes of major public health events, and establish the relationship between cyber-virtual space and geographic real space.

The rest of the paper is organized as follows. We introduce a new model. We discuss event evolution in Section 3.1 and epidemic distribution in Section 3.2. We use the proposed model to perform experiments on two types of data. In Section 3.3 and Section 3.4, network construction and structural analysis were performed, respectively.

## 2. Overview and Data Introduction of the Research Area

### 2.1. Overview of the Research Area

The study area is Wuhan, which is the largest national central city in Central China, and according to the 2018 Wuhan Statistical Yearbook (http://tjj.wuhan.gov.cn/), Wuhan has a total of 13 districts and a resident population of 1121.2 million, of which 7 districts are main urban areas.

**Figure 2:**
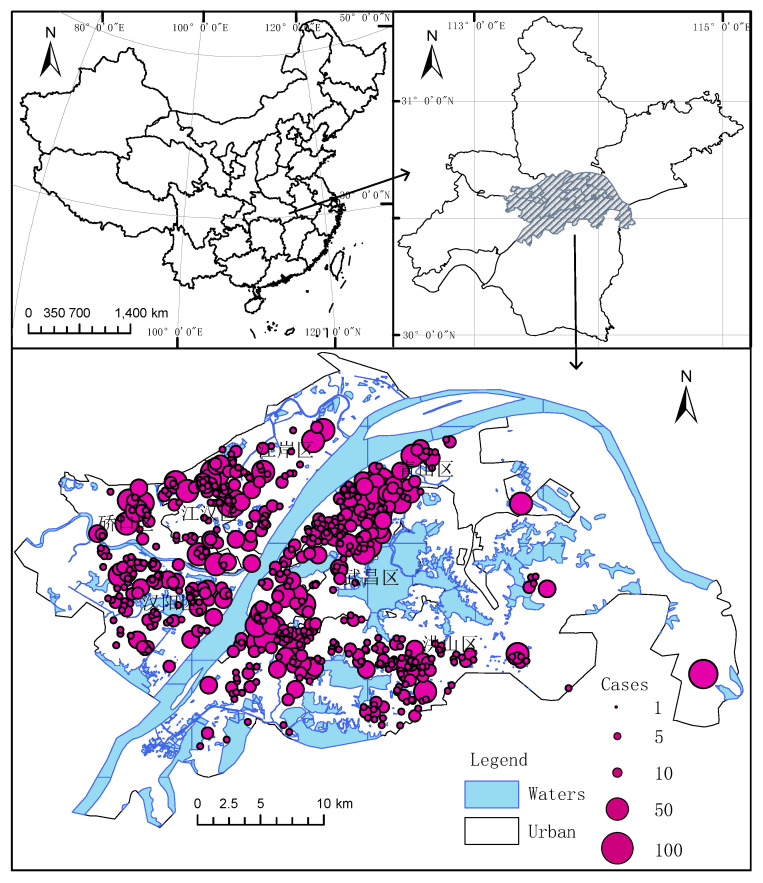
Study area infection data.

The coordinate system of all spatial data in this paper is the China Geodetic Coordinate System 2000 (CGCS2000). We plotted the geographic location of Wuhan in Figure 2.

### 2.2. Data Sources and Preprocessing

There was a lot of discussion about the COVID-19 outbreak during the epidemic. According to the timeline, the growth rate of confirmed cases slowed down on 16 February 2020, the new confirmed cases “fell back” to three digits on 19 February 2020, and on 21 February 2020, 25,000 hospital beds in cubicles have been built, and the shortage of medical resources had been addressed. In the meantime, the State Council Information Office issued that the epidemic had changed positively and the control work had achieved good results (http://cpc.people.com.cn/n1/2020/0221/c431601-31597371.html). This article covers the period from 10 January to 17 February 2020, from the beginning of the epidemic’s development to the beginning of the epidemic’s subsidence, and collects all original Weibo related to the epidemic in the past month.

Based on the Scrapy crawler framework and the Mongodb database, an advanced search script was developed for Sina Weibo, and keywords such as pneumonia, epidemic, and virus were set to increase the amount of retrieved data. After the final search and de-duplication, a total of 213,869 original Weibo data were retained.

The location data contained in the social media data are usually in the form of address descriptions, which we need to geo-decode. Baidu’s address parsing function (http://lbsyun.baidu.com/) is used to process the captured check-in address data and perform spatial location statistical inference and address matching. Due to the discrepancy between Baidu coordinates and the CGCS2000 coordinates used in this paper, which may be up to hundreds of meters, we used coordinate correction (from Baidu coordinate system to CGCS200) to solve this problem. In the end, there were about 30,000 check-in data, excluding points outside the study area and those located in a larger range, and about 10,000 Weibos (Just like the tweets on Twitter) containing locations were retained after filtering, with a total of 1033 check-in locations.

The fever clinic data and community infection data used in this paper were compiled from the CDC, local health committees, government agencies, social media, and the subject network data. The road network and zoning data are from Open Street Map (OSM) (www.openstreetmap.org), all data until 17 February 2020.

## 3. Methods

### 3.1. Directional Distribution and Center of Gravity Shift Models

A common method of measuring the trend of a group of points or areas is to calculate the standard distance in the *x* and *y* directions, respectively. These two measurements can be used to define the axis of an ellipse that contains the distribution of all elements. The ellipse is called a directionally distributed ellipse because the standard deviation of the *x*- and *y*-coordinates is calculated from the mean center to define the axis of the ellipse. Just as the directionality of an element can be felt by plotting it on a map, calculating standard deviation ellipses makes this tendency more explicit, to see if the distribution of elements has a particular direction. Directionally distributed ellipses are calculated as follows.

(1) $$C\;=\left(\begin{array}{cc} \mathrm{var}(x) & \mathrm{cov}(x,y)\\ \mathrm{cov}(y,x) & \mathrm{var}(y) \end{array}\right)=\frac{1}{n}\left(\begin{array}{cc} \sum_{i=1}^{n}{\tilde{x}}_{i}^{2} & \sum_{i=1}^{n}{\tilde{x}}_{i}{\tilde{y}}_{i}\\ \sum_{i=1}^{n}{\tilde{x}}_{i}{\tilde{y}}_{i} & \sum_{i=1}^{n}{\tilde{y}}_{i}^{2} \end{array}\right)$$

where *x* and *y* are the coordinates of element *i*; *n* is the total number of elements. The sample covariance matrix is decomposed into standard form, allowing the matrix to be represented by an intrinsic value and an eigenvector. Thus, the standard deviation of the *x* and *y* axes is.

(2) $$\sigma_{1,2}={\left(\frac{\left(\sum_{i=1}^{n}{\tilde{x}}_{i}^{2}+\sum_{i=1}^{n}{\tilde{y}}_{i}^{2}\right)\pm \sqrt{(\sum_{i=1}^{n}{\tilde{x}}_{i}^{2}-\sum_{i=1}^{n}{\tilde{y}}_{i}^{2}{)}^{2}+4{\left(\sum_{i=1}^{n}{\tilde{x}}_{i}{\tilde{y}}_{i}\right)}^{2}}}{2n}\right)}^{1/2}$$

The variance is measured by an adjustment factor to produce an ellipse containing the desired percentage of data points. When working with one-dimensional data, the 3σ criterion is a common rule of thumb which conveys that a certain percentage of the data values will fall within one, two, and three standard deviations of the mean. In a normal distribution, this means that 68%, 95%, and 99.7% of the data values will fall within one, two, and three standard deviations, respectively. However, when dealing with spatial data of higher dimensions (*x* and *y*), this percentage breakdown is rare. A more applicable rule of thumb, derived from the Rayleigh distribution, states that in a two-dimensional coordinate system (*x* and *y*), a one-standard deviation ellipse will cover approximately 63% of the elements; two standard deviations will cover approximately 98% of the elements; and three standard deviations will cover approximately 99.9% of the elements.

### 3.2. Cluster and Outlier Analysis

The criterion for discriminating between spatial clustering and anomalies is based on the Anselin Local Moran Index [32], which is used to identify local epidemic-opinion hotspots and anomalous regions. In the epidemic-public opinion space, the intensity of public opinion topics and node infection results vary and are correlated with each other in different spaces. A positive index indicates “high-high” or “low-low” clustering of spatial elements, i.e., high or low elements with the same attribute value are adjacent to each other and the element is part of a cluster. A negative value of the partial Moran index indicates “high-low” or “low-high” clustering of spatial elements. Neighboring elements of a spatial element are different values, i.e., the element is an outlier and is divided into a high value “high-low” aggregation surrounded by a low value and a low value “low-high” aggregation surrounded by a high value. The Local Moran Index is defined as follows.

(3) $$I_{j}=\frac{x_{i}-\bar{X}}{S_{i}^{2}}\sum_{j=1,j\neq i}^{n}w_{i,j}\left(x_{j}-\bar{X}\right)$$

where: $x_{i}$ is the attribute of element *i*; $\bar{X}$ is the average value of the corresponding attribute of the element; $w_{i,j}$ represents the spatial weight between elements. In this paper, we will study the regional grids, assigning different grids different definitive data based on geographic location relationships, and determining the spatial weight matrix based on the spatial distances between the grids.

### 3.3. Complex Network Construction Based on the Gravitational Model

We regard a residential community as a “cellular”, and use community infection data to construct the interaction between cells according to the gravitational model. Afterwards, the interaction edges are filtered by a certain threshold value, and the epidemic community are used as the network nodes, and the adjacencies between the filtered nodes constitute the edges of the network, thus forming a more complex network structure. This network structure may even contain most of the information of a real public health event [33]. The connected edges of the network structure imply the changing trends of the epidemic event (e.g., the shift of the geographic focus of infection and discussion), and more valuable information can be extracted from this structure (geographic division of infected communities, evolutionary trends of opinions, and evolutionary trends of sentiments) [34,35].

A complex network can be modeled by using a single diagram $G=\left(V,E\right)$. *V* represents the set of all nodes of a complex network and *E* represents the set of all edges. The mathematical expression for *V* is as follows.

(4) $$V={\left[\begin{array}{ccc} N_{11} & \cdots & N_{1m}\\ \vdots & \ddots & \vdots \\ N_{m1} & \cdots & N_{mm} \end{array}\right]}_{m\times m}$$

where *m* is the determined number of nodes, $N_{i,j}$ represents the degree of connectivity from the *i* spatial node to the *j* spatial node, and the larger $N_{i,j}$ represents the stronger the connectivity between regions.

A typical complex network is composed of several nodes and connected edges with different weights between them, and within the complex network consists of several communities, and the interaction between nodes within the same community can be more frequent than between different communities. In order to construct the edge connections of complex networks, this paper attempts to use the basic gravity model (also known as gravity model and field strength model), which constructs the network edges to represent the mutual induced interactions between the communities.

The gravity model is one of the classical models of geography, based on the laws of geography “all things or phenomena are spatially related, but the connection between things or phenomena close to each other is generally closer than the connection between things or phenomena farther away” [36]. The basic idea can be described simply as: the gravitational force between the two places is proportional to the product of a certain scale quantity between the two places, while inversely proportional to the square of the distance between the two places.

In different application scenarios, the scale of the two places often have different forms of performance. In the field of tourism [37], the scale of two places usually represents the level of tourism supply in the destination and the level of tourism demand in the travel destination; in the spatial and trade connection of two places [38,39], the scale of two places is usually expressed in the form of GDP and total population of two places; in the study of interest recommendation [40], the scale of two places is expressed as the number of sign-ups at the point of interest, etc. In this paper, the number of confirmed visits to the community is used as the scale quantity and use Equation (5) to calculate cell connectivity.

(5) $$A_{{}_{i,j}}=KD_{i}D_{j}/d_{{}_{i,j}}^{2}\;$$

Among them: parameter $A_{i,j}$ represents the degree of connectivity between community *i* and community *j*; parameter *K* is a constant, where it takes the value of 1; *D* represents the confirmed infection data of the cells; and *d* represents the spatial distance between cells.

### 3.4. Disaster Evolutionary Chains and Community Structure Discovery Algorithms

Community structure is an important topological feature of complex networks and the interaction between nodes within the same community will be more frequent than between different communities. In this paper, a modularity Louvain algorithm based on multilevel optimization is used to parse the community structure of complex networks, and it is adopted to detect some small areas with closely connected community or cluster structure, which has the advantages of speed and high efficiency [41]. Using epidemic community data in real space and social media data in virtual space, communities are divided according to two steps: modularity optimization and network aggregation.

Modularity is an evaluation indicator that defines the degree of community structure and is mathematically defined as follows:

(6) $$Q=\frac{1}{2m}\sum_{i,j}\left[A_{i,j}-\frac{k_{i}k_{j}}{2m}\right]\delta \left(C_{i},C_{j}\right)$$

where the degree of connectivity $A_{i,j}$ between communities represents the weight of the edge between the node *i* and the node *j*; $k_{i}$ delegates the degree of the node *i* (the number of bars at the end of the arc plus the number of bars at the beginning of the arc); *m* represents the total number of nodes in the complex network; and $C_{i}$ represents the community with node *i*. The δ function is 1 when $C_{i}=C_{j}$, otherwise it is 0. In the random case, the number of edges between node *i* and node *j* is $\frac{k_{i}k_{j}}{2m}$, the closer the modularity to 1 represents the better community partitioning.

The gravity model formula is substituted into the modularity formula to obtain a geographic propagation model of the evolutionary chain of typical public health events for the fused gravity model-complex network.

(7) $$Q=\frac{1}{2m}\sum_{i.j}\left[KD_{i}D_{j}/d_{{}_{i,j}}^{2}\;-\frac{k_{i}k_{j}}{2m}\right]\delta \left(C_{i},C_{j}\right)$$

**Figure 3:**
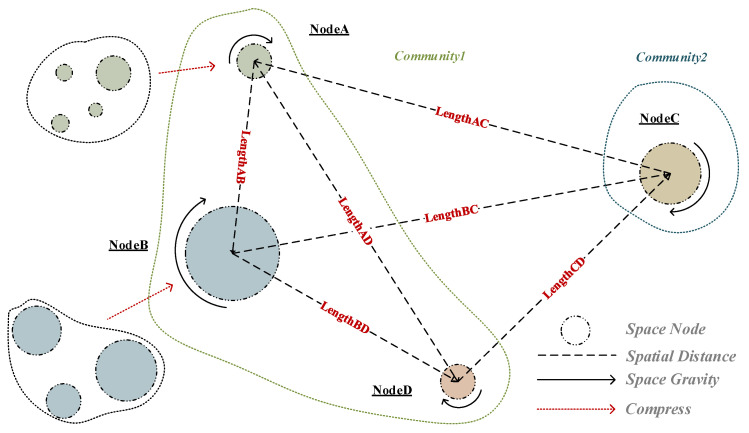
The structure of our proposed model (based gravitational model-complex network).

The model treats each cell as an independent community with the same initial number of communities as the number of nodes. On this basis, all nodes are fused and coalesced based on the modularity gain until the local maximum of modularity is reached, i.e., no node can increase the network modularity, and the community structure is no longer changed. Finally, the community results are compressed by converting their internal node weights into new nodes and weights, and the weights of the original inter-community edges are converted into inter-node weights [42,43]. The algorithm is illustrated in Figure 3.

In addition to the module degree, complex networks have the following more important evaluation metrics.

- Degree of a node: in a directed graph, the number of arc end bars of a node is the out-degree of a node, the number of arc head bars of a node is the in-degree of a node, and thedegreeofanode=out−degree+in−degree. The degree of nodality indicates influence in the epidemic network, where the in-degree indicates the causative event that led to the node’s infection and the out-degree indicates the infection event triggered by the node, the value of them is determined by the system network topology. The greater the node’s out-degree, the more severe the consequences of the node on its neighbors; the greater the in-degree, the more pathways leading to the node, and the more difficult it is to control.
- Betweenness centrality is an indicator of a node’s centrality in a network. It is equal to the number of shortest paths from all vertices to all others that pass through that node. A node with high betweenness centrality has a large influence on the transfer of items through the network, under the assumption that item transfer follows the shortest paths [44].
- Closeness centrality (or closeness) of a node is a measure of centrality in a network, calculated as the reciprocal of the sum of the length of the shortest paths between the node and all other nodes in the graph. Thus, the more central a node is, the closer it is to all other nodes [45].
- A higher PageRank means that the node is more likely to be accessed, and the number of links pointing to the node has a higher link weight [46].
- Average shortest path length reflects the average distance between all nodes and the overall efficiency of the network.
- Average clustering coefficient reflects the average connection tightness of all nodes in the network.
- In complex network diagrams, a higher graph density represents a tighter network connection; a higher degree of modularity represents a more pronounced community structure; a smaller network diameter represents better accessibility between points.

Generally speaking, the greater the degree of outreach of a node indicates that the event has a more widespread impact in the evolutionary chain network of public health events, the more severe the consequences of the event, and is the central node of the evolutionary network of public health events; the greater the degree of inreach of a node, the greater the number of paths of the event, the greater the difficulty of control, and is a key node in the evolutionary development of public health events. The node with the largest betweenness centrality has the strongest connection to the subsequent induced public health event.

## 4. Results

### 4.1. Stages of Public Opinion Development and Shift in Focus

**Figure 4:**
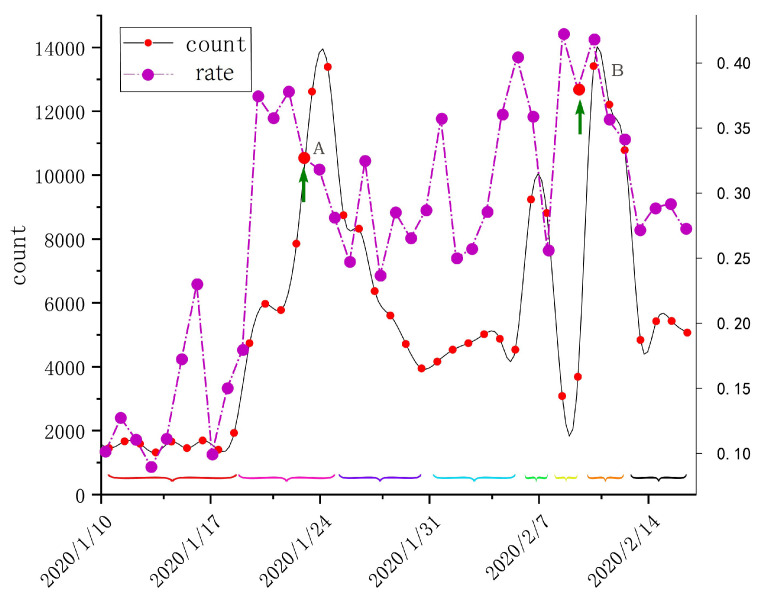
Weibo discussion distribution and emotional change (time A represents the closure of Wuhan, time B represents the start of the full suspected patient intake policy).

As shown in Figure 4, the evolution of public opinion during the influenza pandemic is divided into eight stages based on the timeline between 10 January and 17 February 2020, when more than 200,000 Weibos are displayed according to their temporal attributes.

1. The period from 10 January to 19 January, during which the number of public opinion changes on Weibos was relatively stable,
2. the period from 20 January to 25 January, during which the number of discussions on Weibos increased sharply, can be described as a period of rapid growth in public opinion discussions, which was related to the phenomenon of “human-to-human transmission” as confirmed by academician Zhong Nanshan, followed by the continued fermentation of the pneumonia outbreak. On 23 January 2020 at 10:00 a.m., Wuhan declared to close the city, which is the first time in human history that the toughest epidemic prevention measures have been taken against a major city of 10 million.
3. From 26 January to 31 January, during the Spring Festival, the number of discussions on social media rapidly declined, and the focus of netizens shifted;
4. from 1 February to 6 February, the number of discussions fluctuated, but still at a low level, during this period, the Huoshenshan Hospital delivered and built 11 new hospitals.
5. From 7 February to 8 February, the number of public opinions on Weibo reached a small peak in Wuhan residents’ discussion of the epidemic;
6. from 9 February to 10 February, the heat of the previous discussion had faded, and the medical staff completed the nucleic acid testing of all suspected patients.
7. From 11 February to 13 February, discussions reached another peak due to the closed management of all residential areas;
8. from 14 February to 17 February, related public opinion discussions gradually decreased amid fluctuations. During this period, the closed management of residential areas was further strengthened, and residents’ focus was on the official rumor dispelling information in addition to the epidemic itself.

Based on natural language processing (NLP) techniques, the collected data were categorized into negative, positive, and neutral, and the daily percentage of negative sentiment Weibos were plotted together as shown in Figure 4, where the proportion of negative sentiment reached a maximum of nearly 45% before and after the full suspected patient intake policy was implemented, and then it began to decline.

**Figure 5:**
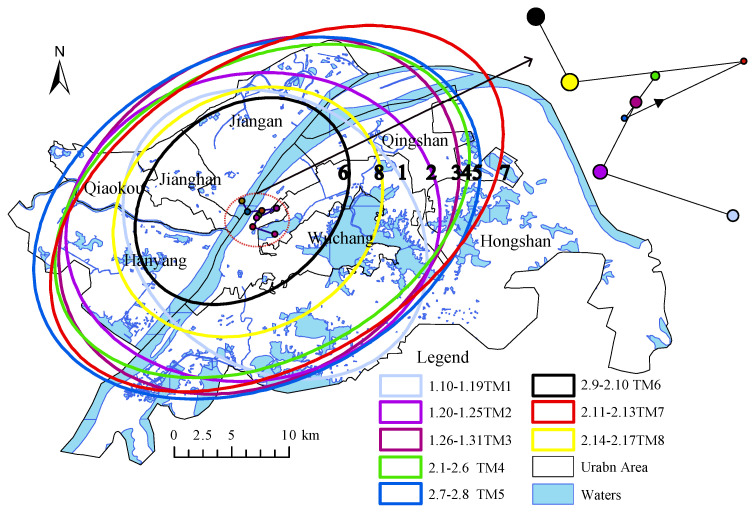
Weibo discussion direction distribution and focus shift.

According to the above-mentioned stages of public opinion evolution, we calculated the 1 standard deviation directional distribution of Weibos in different stages, as shown in Figure 5 and the graph in the upper right corner of it, where the size of the nodes represents the number of check-ins. Stage 1 shows a northwest-southeast directional distribution, with the geographic discussion center in the Sha lake area of Wuchang District; stage 2, the discussion of the climbing period, shows a shift of the geographic center of gravity towards Jianghan District; stages 3, 4, and 5 show a similar directional distribution as the center of gravity; stage 6, the discussion of the trough period, shows a more scattered distribution and a shift of the center of gravity towards the Qingshan District; stages 7 and 8 show a more compact directional distribution, with the geographic center of gravity shifting across the Yangtze River to the other side area. The check-in information in cyberspace is closely related to the population distribution, and an important time point is around 23 January, when the direction is southeast-northwest and then reverses.

### 4.2. Testing with Spatial Anomalies

Since all suspected patients must be seen at the fever clinic, we hypothesized that the commuting distance between the infection node and the fever clinic has an effect on the outcome of the epidemic infection. We first used OSM road network data, 71 fever clinics, and community with confirmed cases for shortest path analysis. The result shows that Shizishan-Zhangjiawan area, East Lake Development Zone-Optics Valley Software Park, and some suburban areas are located greater than 6 km from the fever clinic, with the farthest nodes more than 10 km from the fever clinic.

**Figure 6:**
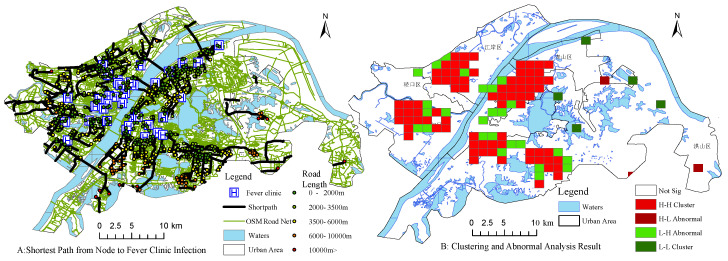
Spatial analysis of the epidemic community. (**A**) Shortest distance and shortest path from node to fever clinic infection. (**B**) Clustering and abnormal analysis (p=0.05 confidence level).

Using the previously mentioned spatial clustering and anomaly testing methods, the main urban area of Wuhan was divided into 30×40 grids, which were processed using the spatial connection tool for the next study. As shown in Figure 6B, the light red grid is the “high-high” clustering area, indicating that there are more confirmed diagnoses in this area and more confirmed diagnoses in the periphery at a confidence level of p=0.05, while the dark red grid indicates the “high-low” area, indicating that there are more confirmed diagnoses in this area and relatively few confirmed diagnoses in the periphery. There are still some “low-high” clustering and “low-low” clustering. The comparison of Figure 6 shows that under certain conditions, the distance to the fever clinic did not have a strong correlation with the confirmed diagnosis as we imagined, and we can reject the original hypothesis.

### 4.3. Geo-Propagation Model of Event Evolution Chain and Complex Network Structure Analysis

**Figure 7:**
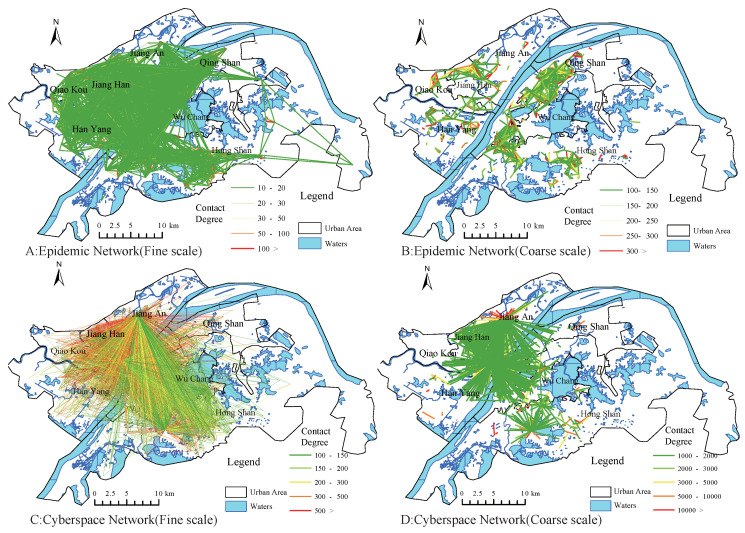
The interactive structure of network reality space in multi-scale.

Based on the gravity model mentioned earlier, we perform complex network modeling of social media data in cyberspace and confirmed outbreak data in real space. In addition, we plot the spatial interactions between nodes at different connectedness scales, as shown in Figure 7. Due to the large number of interaction edges, we filter the network edges with certain thresholds and list the network states with the degree of connectedness greater than 10, the degree of connectedness greater than 100, and the degree of connectedness greater than 1000 after the threshold comparison.

We use a basic hypothesis to deduce that the area with confirmed cases has an impact on the surrounding areas, and this effect increases with the number of confirmed cases, and decreases with the distance between nodes, then the epidemic nodes form a chain of disaster transmission that interacts with each other. In this paper, we use complex network modeling to find the community structure of the epidemic chain. Figure 7A,B is a schematic representation of our geo-spatial interactions based on two different scales constructed for the city cell (with confirmed cases). As a comparison on an opinion dimension, we use the Weibo check-in data to do a comparative experiment, and the results are shown in Figure 7C,D.

**Figure 8:**
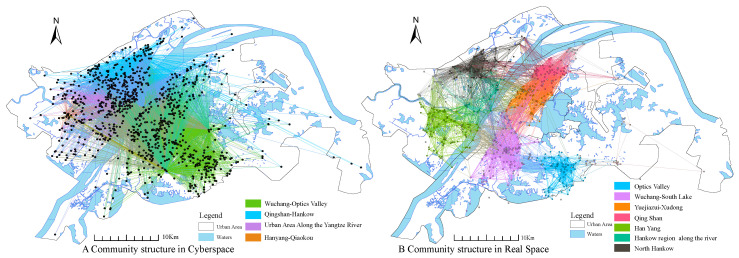
Community division structure of network-reality space.

The results of Louvain’s algorithm are shown in Figure 8, node in real space was divided into seven major communities with very distinct structures, namely, Optics valley, Wuchang-South Lake, Yuejiazui-Xudong, QingShan, HanYang, HanKow region along the river, and North Hankow. The geographical barriers such as Yangtze River and East Lake have a clear watershed effect on the community structure, as opposed to administrative divisions, which do not have significant effects. The influence within the community is much higher than that of the nodes between the communities, and the final division of the modularity is 0.793, showing a strong complex network of community structure.

According to the statistics, seven community nodes have an in-degree higher than 100, indicating that the infection pathways leading to this part of the node are more difficult to control and are key nodes in the evolution and development of the public health event disaster. Four community nodes have an out-degree higher than 100, indicating that these community nodes have the widest impact in this epidemic evolution chain network, the nodes cause the most severe impact, and are the central nodes of the public health event disaster evolution network.

The network structure of the control group as shown in Figure 8B, the community structure along the Yangtze River for segmentation, basically divided into a broad area of Wuhan City along the river, Wuchang-Optics Valley, Qingshan-Hankow, and Hangyang-Qiaokou, the total network modularity of 0.558 less than the real epidemic data network of 0.793. The locations with the largest number of Weibos are Wuhan Union Medical College Hospital, Wuhan Central Hospital and Wuhan Jin-Yin-Tan Hospital, all of which are public designated fever clinics. The number of comments on a single Weibo post at Concordia Hospital was 3138, and such a high level of public attention reflects the heated discussion the epidemic has generated in cyberspace. A Weibo initiative to provide free hotels for medical staff, positioned at Hubei Province Hospital of Traditional Chinese Medicine, has been reposted 3455 times. In addition, a Weibo positioned at Wuhan Union Hospital to cheer up the medical staff has been reposted 2553 times. The epidemic has not only had a huge impact on real space, but has also sparked great concern and discussion in cyberspace, with the large hospital as the frontline of the fight against the epidemic quickly becoming the focus of national attention.

### 4.4. Discussion of Node and Network Characteristics

The density of the complex network graph constructed using the epidemic data was 0.041, higher than 0.024 for the Weibo check-in network, and the average clustering coefficient was 0.7, higher than 0.49 for the Weibo check-in network. Higher modularity of the epidemic network suggests that CVOID-19 could lead to severe localized infections and that there is a possibility of local concentration (localized concentrations) and cross-effects of infection.

In addition, complex networks are also characterized by small world and scale-free [47], with epidemic data networks having small average path lengths (2.962), larger average clustering coefficients (0.7) and more obvious small world effects; point of interest (POI) data networks have relatively smaller average clustering coefficients (0.49). Therefore, it indicates that there is a diversity of interaction behavior in cyberspace and the overall structure of this network is relatively “loose” in general [48,49].

**Figure 9:**
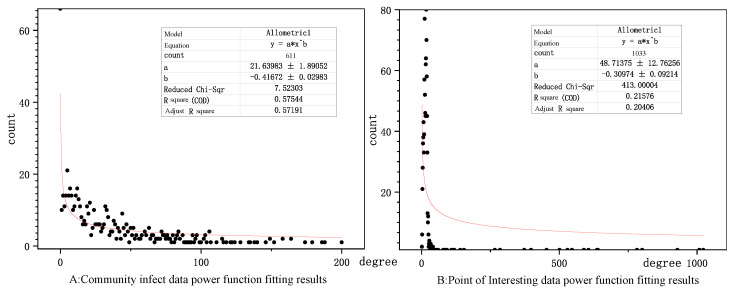
Power-function fit to the degree of a node.

We discuss the scale-free characteristics of the two networks in Figure 9. The scale-free characteristics of the epidemic network are more pronounced, with a fitted $R^{2}$ of 0.572, indicating that except for a few communities with more severe impact, the remaining communities are less infected, and the city closure measures have curbed the spread of the virus in time [50,51]. In contrast, the POI network showed a weaker scale-free feature with a fitted $R^{2}$ of 0.204, indicating that the network information spreads more uniformly in space and has a larger range of influence. Thus, there is no clear indication that Wuhan residents in infected areas focus their attention on certain (few) points of interest.

#### 4.4.1. Spatial Distribution Characteristics of Key Nodes

**Figure 10:**
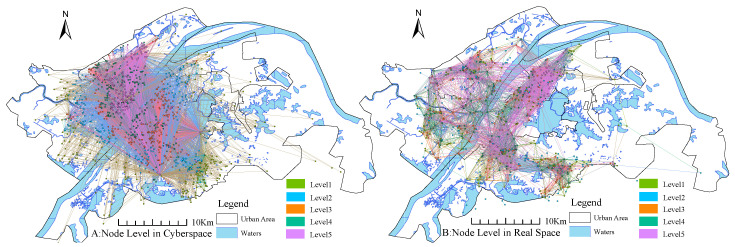
Spatial distribution characteristics of key nodes.

We classify all nodes into 5 levels, from low (Level 1) to high (Level 5), according to their degree values. In Figure 10A, we can see that the distribution of nodes in the POI network is hierarchical, with a large number of Level 5 nodes in the northern part of the Han River and the western part of the Yangtze River, in a decreasing ring shape. The mentioned area is densely populated and affluent, and is a well-known commercial and financial center of Wuhan. In addition, there are a number of POIs, such as the infectious disease hospital, which is the first line of defense against the epidemic, and the South China Seafood Market, where the SARS-CoV-2 virus has been found, which generated a great deal of discussions during the study period. Correspondingly, there are multiple clusters in the real space community network (Figure 10B), and the degree peaks appear in the cluster center to show multi-community distribution. That is to say, there are multiple regions with degree peaks, which also reveals the geographic transmission characteristics of COVID-19, the multicenter distribution is very likely to trigger mass infection.

#### 4.4.2. Risk Assessment of Key Node-Induced Infections

**Figure 11:**
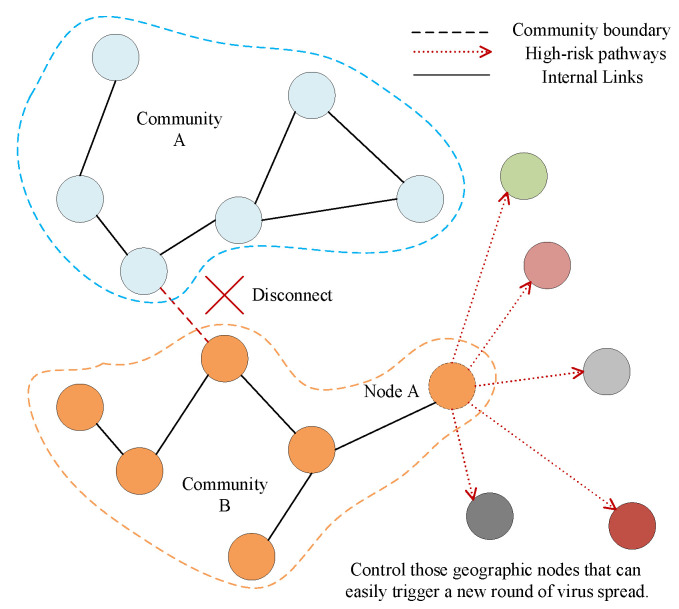
Key nodes open up cross-community spreading paths and induce new rounds of spread.

We mentioned in Section 3.4 that a node with high betweenness centrality has a large influence on the spread of virus through the network. The more nodes with high betweenness centrality, the easier it is for the virus to spread and the faster the number of people diagnosed will rise. So it is an effective way to stop a new round of virus spread through controlling these geographic nodes. In addition, complex networks are tightly connected within communities and sparsely connected between communities, which opens up possibilities for prevention and control. By controlling a few strong connections between communities, we can effectively prevent cross-infection and transmission between communities as shown in Figure 11.

The 30 communities attribute with the highest degree of outbound and inbound for the real space community nodes are in Table 1 and Table 2. We perform calculations and classifications based on node attributes.

We have divided the key nodes into three categories. As shown in Table 2, Shihua Community has high in-degree, but low betweenness centrality, and it is susceptible to the influence of other nodes but has low importance in the network, making it difficult to induce new community infections in the network. Correspondingly, the Tujia Gou node has high out-degree (ninth), very high in-degree (third), and high betweenness centrality, indicating that the node is very well connected throughout the network, and that poor control (untimely response) is highly likely to trigger a new round of virus spread. In addition, there is another type of node that has high out-degree but low betweenness centrality, such as Huateng Park and Baoli City. While there may be more confirmed cases at these nodes, they have a lower risk of spreading across the network.

## 5. Conclusions

This article describes the epidemic situation in the most affected areas of China. From the perspectives of traditional public opinion analysis and geographic location analysis, we explore the migration of the center of gravity of public opinion on social media, and the clustering/anomalies of epidemic communities. More importantly, we propose an innovative model that combines network science and geoscience. The complex network relationships implied by network public opinion data and node infection data are used to construct directed edges, establish network simulation evolution chains in the context of major public safety events, assess the riskiness of disaster nodes, as well as the entire network. Cities are the primary carriers of human economic activity, and the implementation of a total city closure would result in severe economic losses. The virus will likely continue to spread for some time before a vaccine is used on a large scale. Controlling the critical geographic nodes of disease onset would help slow the spread of the virus. Smartphone developers can embed this model into applications that dynamically calculate the riskiness of different regional nodes based on actual epidemics, guiding people to reduce clustering in high-risk nodes. The limitations of our model are that it only uses data from one point in time, and the risk of network nodes should be dynamically simulated over time. It would be an interesting and valuable direction of research to adjust the networks with incorporate concrete application scenarios.

## Figures and Tables

**Table 1:** Community node characteristics of the first 15 out-degrees.

Label		Degree	Pageranks	Clustering	Weighted Degree	Closness Centrality	Betweeness Centrality
Donghu Park	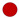	186	0.004754	0.300763	12,686.20319	0.476528	9205.180589
Meilin City	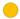	138	0.004209	0.347815	16,319.48388	0.446103	1866.0054
Wutie Jiayuan	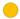	158	0.004872	0.317608	16,395.65837	0.457094	3752.134351
Hankow Park	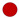	164	0.005962	0.212774	16,608.994	0.477798	11,537.64855
HuaTeng Park	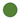	135	0.003562	0.394234	7327.18642	0.423956	962.671439
Baoli City	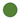	118	0.003749	0.436314	12,921.92453	0.433521	966.821575
Youth City	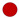	102	0.005272	0.306832	24,364.07104	0.392414	5848.676701
LiujiaWan	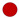	179	0.005157	0.242002	9308.111524	0.481647	9079.850222
Tujia Gou	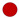	188	0.006043	0.218761	28,789.36615	0.522838	19,417.4878
Jindi City	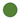	96	0.002979	0.56317	10,283.52847	0.415444	538.549386
Zhongtie Gardens	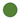	122	0.004012	0.394547	11,707.94896	0.446473	1570.912302
Changhui	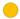	107	0.00426	0.300541	5782.64739	0.437754	2911.917668
Xinyuan	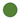	97	0.003154	0.469471	18,890.79875	0.389291	309.077417
East Lake World	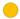	128	0.003599	0.382342	7953.101602	0.452481	3471.562605
Water Land	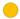	116	0.004507	0.323293	21,928.30106	0.474009	2566.284983

*The risk of inducing disaster*: high level 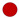; medium level 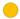; low level 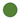.

**Table 2:** Community node characteristics of the first 15 in-degrees.

Label		Degree	Pageranks	Clustering	Weighted Degree	Closness Centrality	Betweeness Centrality
Shihua Community	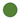	158	0.005106	0.293181	27,739.97498	0.452481	3200.213496
Tangjiadun	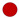	200	0.006903	0.173837	23,380.43404	0.481216	12,506.69791
Tujiagou	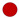	188	0.006043	0.218761	28,789.36615	0.522838	19,417.4878
Garden Community	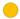	124	0.004718	0.316876	20,042.3545	0.489091	4804.814393
Democratic Road	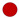	174	0.005566	0.234323	8980.640317	0.491773	8158.937741
Badajia Garden	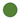	149	0.003735	0.401493	120,230.4021	0.440622	2506.23708
EastGate Community	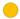	164	0.004989	0.26087	9533.004756	0.476106	4030.957155
Songtao Garden	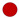	152	0.005291	0.258065	11,764.26325	0.492223	7989.833793
LiujiaWan	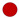	179	0.005157	0.242002	9308.111524	0.481647	9079.850222
Gangdu Garden	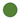	149	0.004185	0.359463	16,996.93004	0.444628	2181.494609
Peace Community	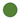	115	0.00362	0.308005	2224.0032	0.452481	1673.350427
Gahua Village Street	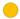	140	0.004398	0.376344	91,838.00845	0.458262	5154.892949
Tongxin Garden	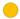	146	0.005264	0.239945	23,130.46086	0.467014	7360.411703
Wuheli Community	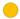	103	0.003893	0.311475	10,964.77266	0.431435	3505.984432
Mei-Yin Temple	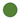	134	0.004719	0.331766	34,497.7463	0.469459	2359.869308

*The risk of inducing disaster*: high level 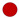; medium level 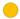; low level 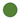.
